# Hand Compartment Syndrome After Prolonged Robotic-Assisted Laparoscopic Rectal Resection: A Case Report and Literature Review

**DOI:** 10.1155/2024/5358112

**Published:** 2024-10-24

**Authors:** Han Y. Zhang, Susan Samudre, Xiuli Zhang, Justin J. Lui, Fenghua Li

**Affiliations:** Department of Anesthesiology, SUNY Upstate Medical University, Syracuse 13210, NY, USA

## Abstract

Hand compartment syndrome is a rare condition due to trauma and vascular obstruction or injury, such as fluid extravasation, vascular obstruction, or arterial injury from arterial line insertion during anesthesia. However, perioperative hand compartment syndrome with no apparent preexisting cause during anesthesia that requires fasciotomy is extremely rare. We report a case on a 54-year-old male with history significant for stage IIIA moderately differentiated adenocarcinoma of the rectum who was scheduled for ultralow rectal resection and cystourethroscopy with insertion of urethral stent. Following surgery, in which he was in a safe-T-Secure positioner in a prolonged Trendelenburg position, the patient developed compartment syndrome of the right hand. This case and literature review highlights the risk for hand compartment syndrome when patients have increased Body Mass Index, positioning involves tucking of the arms, and the procedure duration is prolonged.

## 1. Introduction

Hand compartment syndrome (HCS) is a relatively uncommon condition due to trauma and vascular obstruction or injury [1, 2]. HCS resulting from fluid extravasation, vascular obstruction, and arterial injury from arterial line insertion during anesthesia has been reported [3–6]. However, perioperative HCS with no apparent preexisting cause during anesthesia that requires fasciotomy is extremely rare, with the incidence of 0.6 per 100,000 [7–9]. Overzealous draw sheet tucking of arms has led to the formation of perioperative HCS during surgery [8, 9]. We report a case of HCS following a robotic-assisted laparoscopic rectal resection with safe-T-Secure positioner in a prolonged Trendelenburg position.

## 2. Consent

We obtained a Health Insurance Portability and Accountability Act authorization from the patient to publish this article. This article adheres to the applicable EQUATOR and CARE guidelines.

## 3. Case Description

The patient was a 54-year-old male with history significant for stage IIIA moderately differentiated adenocarcinoma of the rectum who was scheduled for ultralow rectal resection and cystourethroscopy with the insertion of urethral stent. His other past medical history included concurrent radiation and chemotherapy prior to surgery, obesity with a Body Mass Index (BMI) of 37.1 kg/m^2^, as well as a non-ST elevation myocardial infarction (NSTEMI) status post drug-eluting stents to the right coronary artery. His preoperative labs were within normal limits. The patient's American Society of Anesthesiology (ASA) Physical Status Classification was ASA III due to his history of NSTEMI and stents greater than 3 months prior.

On the day of surgery, a 20-gauge peripheral intravenous catheter (PIV) was placed in the right hand in preoperative holding. Anesthesia was induced with propofol (1.5 mg/kg) and fentanyl (1 mcg/kg). Rocuronium (10 mg) was given for defasciculation, followed by intravenous succinylcholine (200 mg) for paralysis during endotracheal intubation. Throughout the case, anesthesia was maintained with sevoflurane. ASA standard monitors were employed. A second 16-gauge PIV was placed in the left hand for improved access and a bladder catheter was used to monitor the urine output. To monitor blood pressure, a radial arterial line was used. Three attempts were made to cannulate the left radial artery for arterial line placement without success, so a pressure dressing was subsequently applied. The right radial artery was easily cannulated in one attempt with a 20-gauge 1-1/4 inch intravenous catheter, which was sutured in place. Arterial wrist support was used to hold the wrist in the extended position.

The patient was positioned in lithotomy on a foam Trendelenburg strap positioner (safe-T-Secure positioner) with arms secured on arm boards less than 90° for the urological portion of the procedure. At completion of the urology procedure, the patient's arms were tucked with the Safe-T-Secure positioner. Of note, the arterial wrist support on the patient's right upper extremity was tucked as well. The patient was placed in steep Trendelenburg with legs in high lithotomy for the duration of the procedure until the bed was leveled approximately 7 hours later.

There was no reported complication intraoperatively. The patient received 3 L of crystalloid, five hundred milliliters of albumin. Estimated blood loss was three hundred milliliters. The patient was hemodynamically stable throughout the case. Total intraoperative time was 13 hours. Because of the duration of the case, the total volume administered, and the potential for airway edema, the decision was made to transport the patient intubated to the surgical intensive care unit (SICU). At that time, no edema was noted in the hands or elsewhere.

The patient was extubated the next morning and he soon complained of pain to his right hand. Orthopedic surgery was consulted. Localized swelling and paresthesia to all nerve distributions of the hand were noted. Both passive and active flexion and extension of the fingers, as well as the wrist, were preserved. Capillary refill was present in less than 2 seconds. At that time, there was low concern for compartment syndrome given the physical exam findings of preserved range of motion and strength. The patient was given instructions to elevate the right upper extremity. However, over the next 2 hours, he experienced worsening pain and edema of the right hand, consistent with the classic “pain out of proportion” description. The only remarkable finding on X-ray of the right hand was diffuse soft tissue swelling. The decision was made to proceed with emergent fasciotomy for acute compartment syndrome and carpal tunnel syndrome of the right hand.

Intraoperative findings noted significant swelling to the right hand with severe compression most notably at the carpal tunnel and thenar eminence. A total of five incisions were made for compartment releases and all wounds were closed except for the thenar eminence incision to allow for swelling. Postoperatively, the patient experienced neuropathic pain and weakness (4/5 strength) with paresthesia in the median nerve distribution of his fingers that improved throughout his hospital stay.

To follow up on his initial rectal resection, the patient underwent a flexible sigmoidoscopy and was noted to have ischemic changes in the anal canal. He returned to the operating room on postoperative day seven for a planned coloanal anastomosis, however, he ultimately underwent an end colostomy secondary to conditions that precluded the coloanal anastomosis. He was discharged home in stable condition although he had deficits in digit opposition and numbness in the first, second, and third digits of the right hand. However, after outpatient physical and occupational therapy, the patient had full recovery of his right hand's motor and sensory function at his 2-year follow-up.

## 4. Discussion

Compartment syndrome is a rare occurrence with serious sequelae that can lead to permanent neurological changes and even cause death without early diagnosis and treatment [1]. Compartment syndrome occurs when there is increased interstitial pressure in a closed space [1]. Increased interstitial pressure may be due to a decrease in the intracompartment size or an increase in fluid volume in the intracompartment space [10, 11]. This increased pressure leads to compromised microcirculation and decreases perfusion of tissue [10, 11]. The diagnosis of compartment syndrome is predominantly based on clinical findings [10, 11]. The clinical findings are characterized by pain out of proportion to the initial injury, diminished sensations secondary to nerve ischemia, and in the late stages, paralysis of muscles within the compartment [10, 11]. Distal pulses are usually preserved unless there is vascular injury. If diagnosis is unable to be determined with clinical findings, pressure measurements in the affected compartments are required. Normal intracompartmental pressures are defined as 0–8 mmHg and critical pressure is defined as greater than 30–40 mmHg [10–14]. Compartment pressures within 30 mmHg of the diastolic pressure are considered diagnostic of compartment syndrome [10–14]. The treatment for compartment syndrome is fasciotomy, which is ideally performed as soon as possible after the diagnosis is established, as decreased perfusion for more than 8 hours is associated with irreversible tissue death [10–14]. The most common cause of compartment syndrome is trauma and most perioperative cases involve the lower extremity [11]. However, in this case, compartment syndrome developed with no apparent preexisting cause in the right upper extremity.

Fourteen cases of HCS were found through a literature review. Direct causes of HCS have been reported to be fluid extravasation, vascular obstruction, and arterial injury from arterial line insertion; these cases are listed on Table 1 [3–6]. However, in our case, there was no sign of intravenous infiltration and although an arterial line was placed, the placement was made in one attempt and without difficulty.  Table 2 lists 10 case reports of HCS that occurred without an apparent preexisting cause [3, 7–9]. Our case is also listed in Table 2. Kies et al. proposed that overzealous uses of the draw sheet to tuck the arms, especially in patients with increased BMI, may increase the risk of HCS by causing a potential tourniquet effect [8]. Comparably, out of the nine cases of that reported patient positioning, six cases involved tucking of the arms. From the other five cases that reported patient BMI, four cases involved patients with BMI greater than 35 kg/m^2^. Eight out of 10 cases reported procedure durations of 250 min or longer. All 10 cases required fasciotomies in the treatment of HCS. Unfortunately, 3 out of 10 of the patients reported significant residual pain and loss of hand function.

Ultimately, our case had many factors in common with other reported cases of HCS, namely, prolonged procedure duration greater than 250 min, patient BMI greater than 35 kg/m^2^, and tucking of the arms for part of the procedure. The use of the Trendelenburg position in addition to the arterial wrist splint may have added additional pressure to the upper extremity. It is difficult to assess and evaluate the upper extremities intraoperatively when they are tucked, as they are obscured by the foam. This patient was also left intubated and sedated when he was transferred to the SICU. Postoperative intubation and sedation might have contributed to delaying early detection of HCS in this patient.

If such conditions are anticipated, care should be taken for patient positioning, not only at the start of the case but also during the case with adjustments as needed. If the patient is kept intubated postoperatively, ICU teams must be reminded to be vigilant of HCS and extubation needs to be done as soon as possible for early detection of HCS. In addition, the careful consideration of the application of arterial wrist support while in use with the safe-T-Secure positioner may be required in the future. The exact cause of HCS is unclear in this case, but these case reports highlight the importance of increased awareness for HCS when patients have increased BMI, positioning involves tucking of the arms, and the procedure duration is prolonged.

## 5. Conclusion

Compartment syndrome is a rare complication with serious sequelae that can lead to permanent neurological changes without early diagnosis and treatment. In this case, compartment syndrome developed with no apparent preexisting cause in the right hand. However, our case did have factors in common with other reported cases of HCS, such as prolonged procedure duration greater than 250 min, patient BMI greater than 35 kg/m^2^, and tucking of the arms during a portion of the procedure. For future cases, if such conditions are anticipated, the care teams must be vigilant of patient positioning, physical exam, and symptoms for early identification and treatment of potential compartment syndrome.

## Figures and Tables

**Table 1:** Case reports of hand compartment syndrome with known causes.

Age (y) and sex (M/F)	BMI (kg/m^2^)	Position	Surgical procedure	Cause	Duration (min)	Fasciotomy	Outcome
38 F	23.4	Prone	Cervical laminectomy, decompressive fusion	Displaced chest roll	200	No	Complete recovery at discharge
34 F	45.8	Supine	Abdominoplasty	IV fluid extravasation	320	Yes	No ischemic findings, no motor or sensory loss at discharge on day 4
44 M	Unk	Unk	Cervical decompression, arthrodesis	Pressured IV bag	Unk	Yes	No loss of range of motion or sensation at 3-months follow-up
16 F	Unk	Unk	Intrabdominal and thoracic procedures	Arterial line	Unk	No	No pain or tenderness to palpation. Neurovascular integrity was preserved. No follow-up listed

Abbreviations: BMI = Body Mass Index, F = female, IV = intravenous, M = male, Unk = unknown.

**Table 2:** Case reports of hand compartment syndrome with unknown causes.

Age (y) and sex (M/F)	BMI (kg/m^2^)	Position	Surgical procedure	Duration (min)	Fasciotomy	Outcome
54 M	37	Lithotomy with arms partially abducted, steep Trendelenburg with arms tucked with Trendelenburg positioner	Rectal resection, cystourethroscopy	780	Yes	Full recovery of sensory and motor function at 2-year follow-up
42 F	Unk	Lithotomy, arms tucked at sides	Laparoscopic gynecological surgery	310	Yes	Continues to recover with near complete recovery of hand function at 3 months
43 M	36	Supine, arms tucked at sides	Colostomy takedown, revision of a complex abdominal scar	300	Yes	Pain at night and with hand use; dysfunction limits lifting or gripping at 10 months
76 F	Unk	Lithotomy, arms tucked	Repair of rectal prolapse	137	Yes	Painless and full hand function by day 30
63 F	Unk	Arms tucked at sides	Left nephrectomy and removal of tumor thrombus from inferior vena cava	343	Yes	Pain-free with full range of motion and sensation to the fingers by 3 months
51 M	39	Arms tucked	Sigmoid colectomy	200	Yes	Complete resolution by 6 months
62 F	28	Unk	Radical hysterectomy	270	Yes	Minor pain and dysfunction by 6 months
34 M	41	Arms tucked	Cystectomy	250	Yes	Complete resolution
56 unk	37	Arms tucked	Retropubic prostatectomy	190	Yes	Persistent discomfort and minor dysfunction
27 F	Unk	Left lateral decubitus	Right hemipelvectomy	480	Yes	Persistent motor and sensory loss at 2 years
36 M	Unk	Left lateral decubitus	Right craniotomy	1920	Yes	Arterial thrombosis; forearm amputation at day 4

Abbreviations: BMI = Body Mass Index, F = Female, M = Male, Unk = unknown.

## Data Availability

The protected health information used to support the findings of this case report and literature review is restricted by the Health Insurance Portability and Accountability Act in order to protect patient privacy. All other data underlying the results are available as part of the article and references. No additional source data are required.

## Nomenclature

HCS: Hand compartment syndrome
BMI: Body Mass Index
ASA: American Society of Anesthesiology
NSTEMI: Non-ST elevation myocardial infarction
PIV: Peripheral intravenous catheter
SICU: Surgical intensive care unit
